# The floral biology and the role of staminal connective appendages during pollination of the endoparasite *Bdallophytum americanum* (Cytinaceae)

**DOI:** 10.1007/s10265-023-01466-4

**Published:** 2023-06-14

**Authors:** Sandra Rios-Carrasco, Daniel Sánchez, Pactli F. Ortega-González, Morayna F. Gutiérrez-Luna, Manuel Edday Farfán-Beltrán, María C. Mandujano, Sonia Vázquez-Santana

**Affiliations:** 1 Laboratorio de Desarrollo en Plantas, Departamento de Biología Comparada, Facultad de Ciencias, Universidad Nacional Autónoma de México, 04510 Ciudad de Mexico, México; 2 Posgrado en Ciencias Biológicas, Universidad Nacional Autónoma de México, 04510 Ciudad de Mexico, México; 3 CONACYT–Laboratorio Nacional de Identificación y Caracterización Vegetal, Departamento de Botánica y Zoología, Centro Universitario de Ciencias Biológicas y Agropecuarias, Universidad de Guadalajara, 44171 Zapopan, Jalisco México; 4 Posgrado en Ciencias Biológicas, Instituto de Ecología, Universidad Nacional Autónoma de México, Coyoacán, 04510 Ciudad de Mexico, México; 5 Laboratorio de Genética y Ecología, Departamento de Ecología de la Biodiversidad, Instituto de Ecología, Universidad Nacional Autónoma de México, UNAM, 04510 Ciudad de Mexico, México

**Keywords:** *Copestylum*, Floral movements, Hoverfly pollination, Landing platforms, Sapromyophily, Staminal appendages

## Abstract

*Bdallophytum americanum* (Cytinaceae) is an endoparasitic plant species, meaning only the flowers emerge from the host during the reproductive season. Reports on the pollination biology of this species state that its primary pollinators are carrion flies attracted by the smell of the flowers and nectar as a reward. However, the functional role of one of the most outstanding attributes of *B. americanum* has been neglected. These are the staminal appendages formed by the apical overgrowth of connective tissue during anther development. To determine whether these staminal appendages play a role in pollination, we monitored a nectarless population of *B. americanum*. We described the inflorescence emergence, floral movements, and pollination and performed field experiments to test whether the absence of the staminal connective appendages affected the visitation frequency. Male inflorescences emerge early, and both male and female flowers open during the day and do not close. Hoverflies are the most frequent visitors to both floral sexes and carry the most pollen. Moreover, the movement of staminal appendages matching the pollen viability changes is reported for the first time. The staminal appendages are the structures where pollinators land before foraging. The field experiments showed that the visitation frequency decreased sharply without staminal appendages. As a landing platform, the staminal connective appendages in *B. americanum* are crucial for pollinator positioning and collecting viable pollen.

## Introduction

Parasitic plants obtain their resources from other plants through a specialized structure called the haustorium and have evolved 12 times during angiosperm evolution (Nickrent 2020; Twyford 2018). Parasitic plants can be classified as hemiparasites or holoparasites depending on their photosynthetic capacity. The former retains photosynthetic activity while the latter has lost all photosynthetic functions (Heide-Jørgensen 2008). A subset of holoparasites is classified as endoparasites because their vegetative body grows inside the host, and only the flowers emerge from the host (Teixeira-Costa et al. 2021; Thorogood et al. 2021). The endophytic holoparasites comprise four families from different orders: Apodanthaceae (Cucurbitales), Cytinaceae (Malvales), Mitrastemonaceae (Ericales), and Rafflesiaceae (Malpighiales), the latter being the most notable species within the parasitic angiosperms (Nickrent 2020; Thorogood et al. 2021). Since the species of these families externally comprise only flowers or inflorescences, floral biology studies are essential to understand part of the life cycle of these peculiar species.

Floral biology ranges from the flowers’ emergence, form, and function to advertisements and rewards concerning pollination (Gottsberger 1989; Willmer 2011). Pollination has been studied in some endoparasites, and due to their peculiar morphologies, the study of the form and function of the flowers has become important to understand aspects of the reproductive biology of the species (Bänziger 1991; Beaman et al. 1988; De Vega et al. 2009; García-Franco and Rico-Gray 1997; Hobbhahn and Johnson 2015; Johnson et al. 2011; Sipes et al. 2014; Suetsugu 2019). Within these endoparasites, the Rafflesiaceae species are the most studied, and their flowers have unique traits whose function has been related to carrion fly pollination (Faegri and van der Pijl 1979; Nikolov et al. 2014). An example is the presence of a barrier comprising floral acicular hairs allowing the entry of pollinators (Bänziger 1995). This prevents pollen robbery by non-pollinators, and the stigma form facilitates pollen deposition in Rafflesiaceae flowers (Beaman et al. 1988). Filiform appendages have been related to fly-pollination serving as landing platforms or scent-emitting structures, which influence the attraction of pollinators and their behaviour (Katsuhara et al. 2017; Suetsugu et al. 2021, 2022). In addition to filiform appendages, the strong foul smell, large or small flowers clustered in inflorescences, in some cases the presence of nectar, colours resembling decaying meat or dung, and the presence of hairy pads are some of the features that characterize the sapromyophilous pollination syndrome (Faegri and van der Pijl 1979; Moir et al. 2022; Shuttleworth et al. 2017; Suetsugu et al. 2022; Willmer 2011).

Sapromyophily has been highly reported for different non-parasitic angiosperm species such as Araceae, Apocynaceae, Aristolochiaceae, Orchidaceae, and others (Johnson 2016; Jürgens and Shuttleworth 2015), but as we mentioned before, the sapromyophilous endoparasites have received less attention. In that sense, in addition to Rafflesiaceae, other endoparasitic sapromyophilous species exist, such as those of the genus *Bdallophytum* of the Cytinaceae family (García-Franco and Rico-Gray 1997; Rios-Carrasco et al. 2022a, 2022b). Pollination studies confirmed that *B. americanum* (formerly *B. bambusarum*) is pollinated by carrion flies (García-Franco and Rico-Gray 1997), while *B. andrieuxii* and *B. oxylepis* are pollinated by butterflies and stingless bees respectively, indicating that carrion behaviour is not exclusive to carrion flies (Rios-Carrasco et al. 2022a, 2022b).

Cytinaceae is the second species-rich endoparasite family, following Rafflesiaceae, with 12 species in two genera (Nickrent 2020). The genus *Cytinus* encompasses eight species from the Mediterranean, South Africa, and Madagascar (Sanjust and Rinaldi 2021), and *Bdallophytum* includes four species from Mexico to Colombia (Nickrent 2020). The first pollination study in Cytinaceae was conducted in *B. americanum* in a population from a tropical semi-humid area (García-Franco and Rico-Gray 1997). However, this species is widely distributed in seasonal vegetation types characterized by marked dry and wet seasons (Alvarado-Cárdenas 2009). So far, the variation in pollination is unknown for other populations of *B. americanum* in different environments. Regarding floral traits, *B. americanum* is a dioecious species whose male flowers are noticeable because they have conspicuous staminal connective appendages resembling a big multilobed stigma. The growth of the apical connective tissue forms these appendages during flower development, creating long extensions, one per anther, that are not seen in other species of the genus (Rios-Carrasco and Vázquez-Santana 2021). The form-function relationship of the connective appendages on male flowers of *B. americanum* remains unknown. In order to know if staminal appendages of *B. americanum* male flowers play a role in pollination as reported for other flowers with appendages, this work aimed to study the floral biology of *B. americanum*, addressing the floral biology and pollination. We especially focus on the functionality of the staminal connective appendages during pollination.

## Materials and methods

### Species and study site

**Fig. 1:**
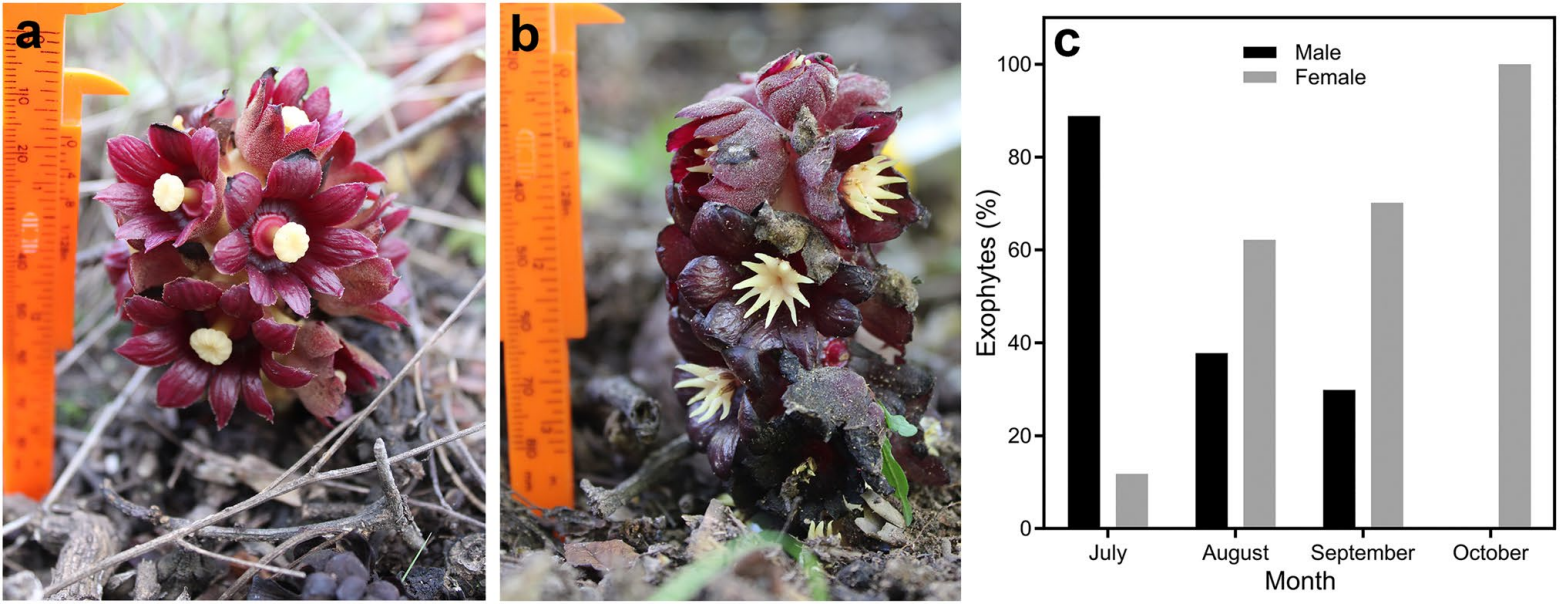
Exophytes of *Bdallophytum americanum*. **a** Female inflorescences (exophytes). **b** Male inflorescences (exophytes). **c** Percentage of emerged exophytes of *B. americanum* per sex through time in the population of Calvillo, Aguascalientes, Mexico. The withered male inflorescences were not counted

*Bdallophytum americanum* Harms is a dioecious endophytic holoparasite species belonging to Cytinaceae. This dioecious species is widely distributed, with populations from Mexico to Costa Rica (Alvarado-Cárdenas 2009). The species has the fewest flowers per inflorescence (12–18) and the largest flowers within the genus (Rios-Carrasco and Vázquez-Santana 2021). Based on the floral morph, the flowers have a dark purple perigone contrasting with the anthers or stigma. Male flowers have conspicuous bright yellow staminal connective appendages, and the female ones have a bright yellow stigma (Fig. 1a, b). Since each inflorescence emerges from a different site of the host root, here we consider that an inflorescence corresponds to an exophyte, which in turn corresponds to an individual.

The fieldwork was conducted in the municipality of Calvillo in the state of Aguascalientes, Mexico. The weather is predominantly semi-warm, with an average annual temperature of 16.6–20.3 °C and a total annual precipitation of 612.2 mm (Instituto Nacional de Estadística y Geografía 2017). The locality is in a remnant patch of a seasonally dry tropical forest where some species such as *Albizia plurijuga*, *Conzattia multiflora, Lysiloma* spp., *Leucaena* spp. (Fabaceae), *Bursera fagaroides* (Burseraceae), *Myrtillocactus geometrizans*, *Stenocereus queretaroensis*, and *S. drummondii* (Cactaceae) are dominant in the area (Siqueiros-Delgado et al. 2016). Within the municipality, the original vegetation is scarce since fields of guava cultivation have replaced it. This population of *B. americanum* is parasitizing *B. fagaroides,* and we considered each parasitized host as a cluster. The specimen vouchers were deposited at the Herbarium Luz María Villarreal de Puga of the Universidad de Guadalajara as follows: *Sánchez 681* IBUG (male inflorescences of *B. americanum*), *Sánchez 682* IBUG (female inflorescences of *B. americanum*), and *Sánchez 679* IBUG (host *Bursera fagaroides*).

### *Bdallophytum americanum* population

During three flowering seasons from 2018 to 2020, we counted the number of *B. americanum* hosts within 1 km^2^ in a remnant patch of seasonally dry tropical forest between cultivated areas. We counted the number of exophytes on roots for each host (equal to one cluster) and classified them according to sex. It is noteworthy that neither sexual morph showed any secretions or nectar during the fieldwork at any moment or year of study. Thus, this is a nectarless population.

### Floral movements, stigmatic receptivity, and pollen viability

We observed at least 50 pre-anthetic flowers per sex (belonging to 10 inflorescences per sex) to detect any change or movement during opening, as well as the moment of anther dehiscence and movement in the connective appendages throughout the flower lifespan in male flowers. Additionally, we used hydrogen peroxide to determine the moment when stigmatic receptivity begins and its duration (Galen and Plowright 1987). We tagged and bagged 10 female inflorescences to avoid pollen interference on stigmas. At least 15 flowers per age were used to measure stigmatic receptivity in newly opened flowers in 1-, 2-, 3-, and 4-day-old flowers; 30 µL syringes were used to place a few drops of hydrogen peroxide on the stigmas.

Regarding male flowers, we collected pollen samples from different anthers from each flower with a dissection needle. We placed them on glass slides to stain the pollen grains with Alexander’s reagent (Alexander 1980) to determine the duration of pollen viability over time. We classified flowers according to their age, and we calculated the percentage of viable pollen in flowers of different ages (1- to 6-day-old flowers). We used at least 15 flowers per age.

### Floral visitors

We recorded the floral visitors in two flowering seasons during August and September of 2019 and 2020, respectively, for 4 days each year in six clusters (three clusters per sex). Each cluster had at least five flowering inflorescences of *B. americanum*. The records of diurnal floral visitors were produced through direct observations in five inflorescences per cluster from 9:00 to 16:00 h in 15 min observations followed by 15 min of rest. We also used trap cameras programmed in two video modes, (1) to record a one-minute video every 15 min (to detect small visitors) and (2) we activated the motion sensor (to detect larger visitors); both modes were active from 16:00 to 9:00 h. We obtained the number of visitors per inflorescence (male or female), the visited flowers, the foraging time, and the number of visited inflorescences per visitor. We used lethal chambers to capture samples of floral visitors. Mounted specimens were identified at least at the family level and to the finest level possible, using the following keys: de Carvalho et al. (2003), Elberg et al. (2009), Engel (2000), Goulet and Huber (1993), Hernández et al. (2013), Knutson and Orth (2001), Thompson (1999) and Triplehorn and Johnson (2005). When the species level was not reached, we used the morphospecies criterion.

We classified the floral visitors into functional groups according to their foraging behaviour (Fenster et al. 2004). Particularly, we split the fly functional group into two according to the foraging behaviour we observed in the field. The first group was treated as “flies” and the second as “hoverflies”, given their pollinivorous and landing behaviour described in the results section. To determine differences in the visitation frequency among functional groups, we performed a generalized linear model (GLM) with a Poisson distribution and log-linkage function using the package *stat* in the software R (R Development Core Team 2019). We used the visitation frequency as the response variable and the functional groups, years of observation, and sexes as the explanatory variables. Then, a Chi-square test was performed to examine if differences existed between each explanatory variable. Additionally, we performed a multiple comparison analysis of the visitation frequency between functional groups. We considered pollinators those that visited female and male inflorescences, consistently touching the sexual organs and carrying pollen from donors on their bodies. Visitors who approached only one sex or did not touch sexual organs were discarded as pollinators.

To identify the functional group that contributes the most to pollination, we evaluated the pollination important index (PII) following Lindsey (1984) and Mochizuki and Kawakita (2018). We calculated the PII considering (1) the relative abundance expressed as the proportion of the visitors collected per functional group (A) of each functional group; (2) the pollen carrying capacity (PCC) expressed as the average of pollen load; (3) relative host fidelity (F) expressed as the proportion of carried host pollen, and (4) pollinator effectiveness (PE) which indicates the probability of a visitor to pollinate. Following Mochizuki and Kawakita (2018), we assigned a PE value of 1 if the visitors (belonging to the same species or morphospecies) always touched both sexual organs (indicating that they visited both male and female inflorescences), and 0 if the same visitor species or morphospecies touched only one sexual organ (only visited one inflorescence sex) or none. As some visitors occasionally touched both sexual organs, we assigned them a PE value of 0.5. We estimate the pollination importance value (PIV) for each functional group through the following equation: PIV = A × PCC × F × PE. Then, to obtain the PII per functional group, including both years of study, we used: PII = PIV∕ƩPIV (Lindsey 1984; Mochizuki and Kawakita 2018). We excluded functional groups whose visit frequency was so low that it was not possible to capture specimens.

### Role of connective appendages in pollination

We performed a field experiment in 2020 to explore the role of the staminal connective appendages by evaluating whether their absence affected the visitation frequency of pollinators to male inflorescences. The experiment comprised two floral conditions, the mutilated and the intact flowers. In the first, the apical connective appendages were removed from all flowers of an inflorescence. In the intact, the flowers were not handled, serving as a control. Both treatments were applied to all inflorescences from two clusters namely pure clusters since all the flowers presented the same condition, one cluster with only mutilated flowers (*n* = 10 inflorescences), and in the second only intact flowers (*n* = 10 inflorescences). To discard the cluster effect and evaluate the decision-making by floral visitors, we randomly applied both treatments (mutilated and intact) into a single cluster, namely the mixed cluster (*n* = 10 inflorescences, 5 per treatment). The visitation frequency was recorded following the same method described above for floral visitors. We compared the total visitation frequency (response variable) between treatments (mutilated *vs* intact) and between cluster types (pure *vs* mixed). The comparisons were performed through a GLM with a Poisson distribution, log-linkage function, and chi-square to determine differences between treatments and clusters using the software R Development Core Team (2019). The analysis was conducted considering a daytime observation time from 9:00 to 19:00 h to avoid data overdispersion since the pollinators are diurnal.

## Results

### *Bdallophytum americanum* population

The studied population of *Bdallophytum americanum* comprised nine clusters within approximately 1 km^2^ in a seasonally dry tropical forest patch. During the first year of monitoring (2018), we found six clusters, of which three had exophytes from that season, while the other three had remains of exophytes apparently from the previous season. Moreover, the same clusters were found in the next two years, and three clusters more during the last year. Most exophytes had the same sex within a cluster, and this was maintained through the years (Table 1), indicating re-emergence.

**Table 1:** Number and sex of exophytes per cluster (host) of *B. americanum* indicating reemergence through years in the population of Calvillo, Aguascalientes

Cluster	Number of exophytes and their sex		
	2018	2019	2020
1	47 ♂	47 ♂	40 ♂
2	4 ♀, 13♂	38 ♀, 8 ♂	41 ♀, 10 ♂
3	10 ♀	17 ♀	11 ♀
4	14 ♀	13 ♀	14 ♀
5	24 ♀	30 ♀	25 ♀
6	41 ♂	33 ♂	40 ♂
7	–	–	10 ♂
8	–	–	2 ♀
9	–	–	4 ♀

The emergence of the inflorescences begins after the first rains in the area. The exophytes or young inflorescences can be observed from July, and as time passes, the number of exophytes increases. The first exophytes to emerge in the studied population corresponded to the male inflorescences, which predominated during the first month, mainly flower buds. The female inflorescences predominated from August to September, while only infructescences were present in October (Fig. 1). Despite the asynchrony at emergence, female and male inflorescences emit a strong unpleasant smell when the flowers are open. This smell is easily detectable by the human nose when close to a patch.

### Floral movements, stigmatic receptivity, and pollen viability

**Fig. 2:**
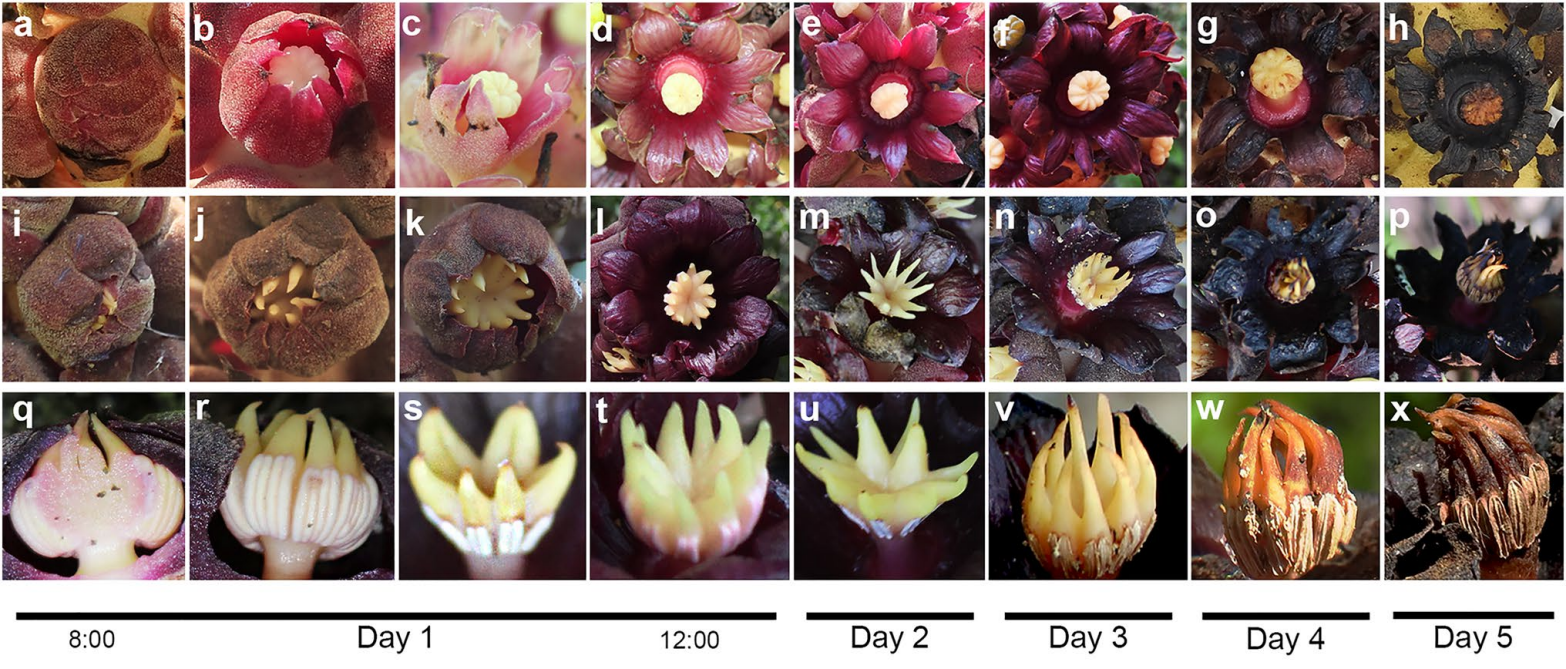
Changes and movements through time in *B. americanum* flowers. **a**–**h** Female flowers showing their colour change since their opening until their withering. **i**–**p** Male flowers opening. **q**–**x** Movements and changes of connective appendages of stamens

During the morning, the flowers are closed (Fig. 2a, i). The flower opening occurs around 8:00 to 9:00 h in the morning (Fig. 2b–c), reaching a full aperture at approximately noon (Fig. 2d, l). As the days pass, female flowers exhibit colour changes; they are initially pale red (Fig. 2b–d) and turn darker (Fig. 2e–g). The perigone remains open and withers around day 5 (Fig. 2h), indicating the start of fruit development if fertilization has occurred. In male flowers, the perigone is dark red from the beginning (Fig. 2i–k). Female flowers reach the full aperture at noon (Fig. 2l); the flowers remain open until they wither on day 4 or 5 (Fig. 2m–o), later appearing whiter (Fig. 2p). In the male flowers, the anthers and appendages of the connective tissue also showed movements and changes along the flower lifespan (Fig. 2q–x). The apical connective appendages are towards the centre when the flowers open, so the tips touch each other and protrude above the perigone (Fig. 2q, r). The appendages then extend out as the perigone unfolds to achieve a complete opening (Fig. 2s–u). The anther dehiscence is extrorse. This occurs when the flowers are completely open, and the connective appendages are fully extended (Fig. 2t, u). The appendages remain extended during the first 2 days of anthesis (Fig. 2t, u). The appendages begin to darken and retract towards their initial position on the third day (Fig. 2v–x).

**Fig. 3:**
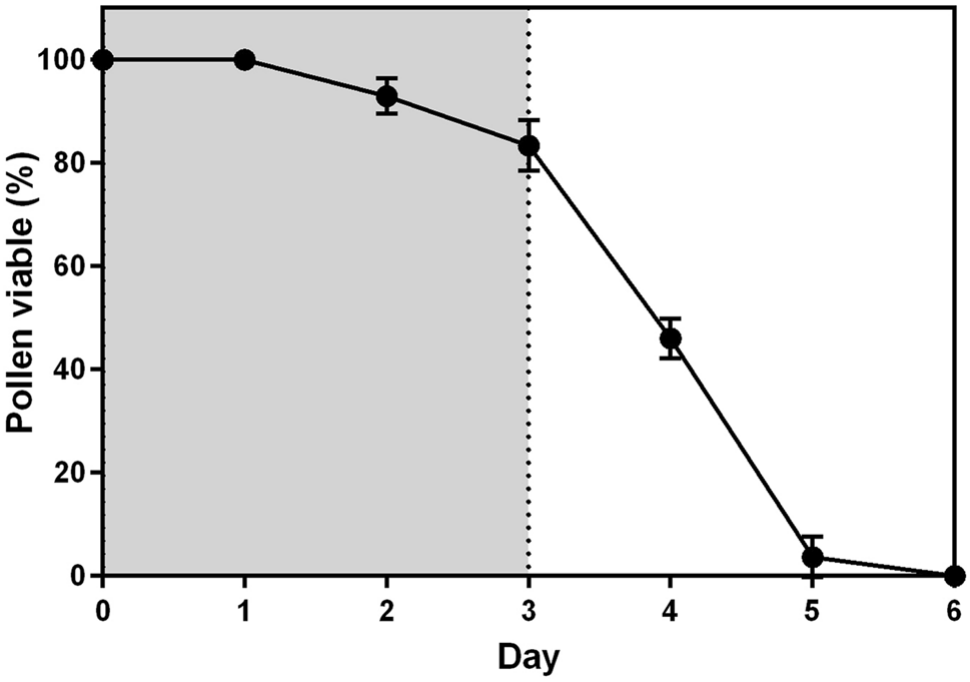
Decreasing in viability of pollen grains in male flowers of *B. americanum* over the days. The gray area indicates the duration of stigmatic receptivity in female flowers

Regarding sexual functionality, the pollen grains are viable in male flowers during the first 4 or 5 days. Nevertheless, from the third day, viability decreases abruptly. The pollen grains are no longer viable on the sixth day (Fig. 3). The stigmas in female flowers start to be receptive as soon as the flowers start to open. The receptivity lasts 3 days. The stigmas are no longer receptive on day 4 (Fig. 3).

### Floral visitors

**Fig. 4:**
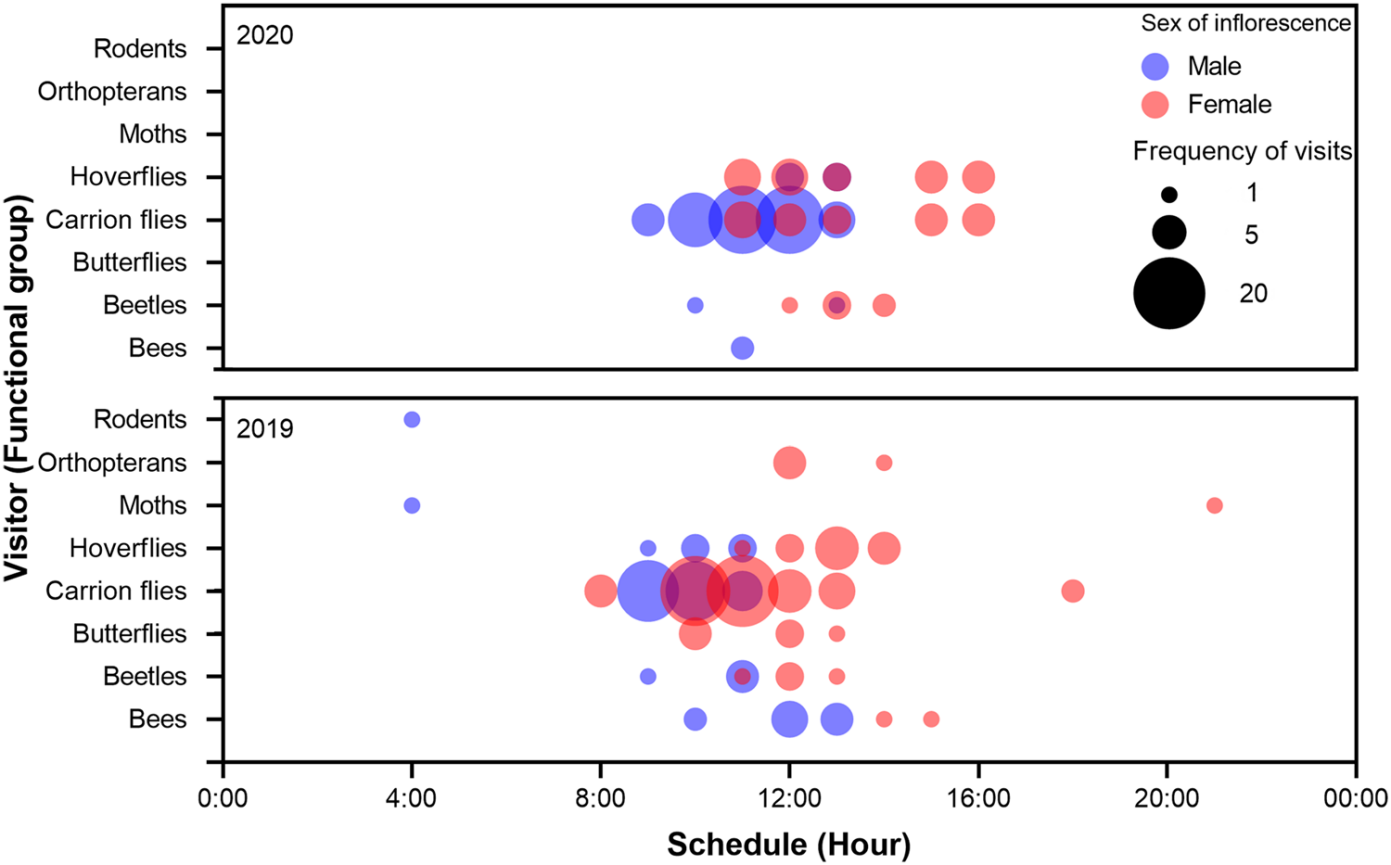
Floral visitors to *B. americanum* by functional groups to see the frequency of visits to male and female inflorescences along the day in both years of study. Potential pollinators can be distinguished by overlapping different coloured bubbles

We recorded the visits of two species and 12 different morphospecies belonging to eight functional groups (Table 2) in a total of 2052 min of observation during both years of study. Most visits were recorded in a diurnal schedule and only rodents and moths were rarely seen at night-time (Fig. 4). Male inflorescences had more frequent visits than female inflorescences (χ^2^ = 6.166, df = 1, *p* < 0.0001). Additionally, we registered more visits in 2019 from more functional groups than in 2020 (χ^2^ = 14.156, df = 1, *p* < 0.0001; see Fig. 4). Regarding the visitation frequency, differences occurred between the functional groups (χ^2^ = 294.431, df = 7, *p* < 0.0001; Table 3). Hoverflies were the most important pollinators in both years of study. Their PII was the highest value compared to other important potential pollinators such as bees, beetles, and flies (see Table 4).

**Table 2:** Species or morphospecies of floral visitors recorded for male and female inflorescences of *Bdallophytum americanum* in Calvillo, Aguascalientes, Mexico during 2019 and 2020

Visitors to female inflorescences			Visitors to male inflorescences		
Functional group	Taxonomical family	Visitor	Functional group	Taxonomical family	Visitor
Bees	Hallictidae	**Augochlorini sp. 1**	Bees	Hallictidae	**Augochlorini sp. 1**
Beetles	Curculionidae	***Epimechus adspersus***			Augochlorini sp. 2
Butterflies	–	Butterfly 1	Beetles	Curculionidae	***Epimechus adspersus***
Flies	Heleomyzidae	***Heleomyzidae* sp. 1**		Nitidulidae	Nitidulidae sp. 1
	Sciomyzidae	***Sepedon* sp. 1**			Nitidulidae sp. 2
Hoverflies	Syrphidae	***Copestylum* sp. 1**			Nitidulidaae sp. 3
Moths	–	**Moth 1**	Flies	Fannidae	*Fannia canicularis*
Orthopterans	Acrididae	Acrididae sp. 1		Heleomyzidae	***Heleomyzidae* sp. 1**
				Sciomyzidae	***Sepedon* sp. 1**
			Hoverflies	Syrphidae	***Copestylum* sp.1**
			Moths	–	**Moth 1**
			Rodents	–	Rodent 1

The visitors were classified into functional groups based on their behaviour. Visitors recorded on both inflorescences are in bold

**Table 3:** Characteristics of the visits from different functional groups to the male and female inflorescences of *B. americanum* in Calvillo, Aguascalientes, Mexico during both years of study

	Female inflorescences				Male inflorescences			
Functional group	Visited inflorescences per cluster Mean ± SEM	Visited flowers per inflorescence Mean ± SEM	Handling time per visit s	Total records	Visited inflorescences per cluster Mean ± SEM	Visited flowers per inflorescence Mean ± SEM	Handling time per visit s	Total records
Bees^ac^	1.5 ± 0.28	1	7.5 ± 2.5	2	2.12 ± 0.35	2.9 ± 0.64	19 ± 5.56	13
Beetles^bc^	1.4 ± 0.4	2.14 ± 0.4	17.44 ± 4.6	11	1.22 ± 0.22	1.42 ± 0.29	41 ± 23.61	7
Butterflies^ac^	1.33 ± 0.33	1.66 ± 0.67	6.55 ± 1.3	8	–	–	–	0
Flies^c^	1.2 ± 0.13	2.27 ± 0.33	34.8 ± 9.6	36	1	1.57 ± 0.29	132.5 ± 25.87	13
Hoverflies^d^	2.12 ± 0.24	3.7 ± 0.48	25.7 ± 4.49	75	1.72 ± 0.23	3.91 ± 0.57	80.86 ± 16.4	87
Moths^a^	1	1	2	1	1	1	4	1
Orthopterans^ac^	1	1.5 ± 0.5	212 ± 66	5	–	–	–	0
Rodents^ab^	–	–	–	0	2	1.5 ± 0.5	4.33 ± 0.3	1

Superscripts indicate differences in the visitation frequency between functional groups resulting from comparison multiple analysis at *p* < 0.001

**Table 4:** Importance of functional groups as pollinators of *B. americanum* in Calvillo, Aguascalientes, Mexico during the two years of study

Functional group	Visitors captured	A	PCC	F	PE	PIV	PII
Bee	4	0.15	17,500	0.7	0.5	907.407	**0.3**
Beetles	5	0.19	370	1	1	68.518	**0.02**
Flies	3	0.11	375	1	0.5	20.833	**0.01**
Hoverflies	15	0.56	3680	1	1	2044.444	**0.67**

*PII * values are in bold

*A* relative visitor abundance, *PCC* pollen carrying capacity, *F* relative plant fidelity, *PE* pollinator effectiveness, *PIV* pollination importance value, *PII* pollination importance index

Regarding the behaviour of visitors, butterflies, moths, orthopterans, and rodents were discarded as pollinators. Most of them only visited one type of inflorescence, or their visits were rare, and they spent a few seconds foraging (Table 3). Additionally, the orthopterans spent a long time eating floral parts. Thus, they were florivorous rather than pollinators. The halictid bees of the tribe Augochlorini (Augochlorini sp. 1 and sp. 2) were the visitors carrying the largest number of pollen grains of *B. americanum.* However, only Augochlorini sp. 1 visited both sexes, meaning that only this bee species could contribute to pollination. On their visits, the bees spent more time collecting pollen than on their visits to female flowers. Besides, the pollen found on the bees’ bodies was not exclusive to *B. americanum* (Table 4). Despite the low visitation frequency to female flowers, the PII of bees was above that of beetles and flies; thus, bees may contribute to pollination due to their high pollen loads. Beetles of the species *Epimechus adspersus* (Curculionidae) were registered to visit both floral sexes. Although beetles spend more time resting in the perigone than touching the sexual organs, they can pollinate as they arrive at stigmas with *B. americanum* pollen. Flies were more frequent than beetles (Fig. 4). They occasionally touched the sexual organs and spent more time resting on the perigone (Table 3). We found that *Sepedon* sp. 1 flies reached the female flowers with *B. americanum* pollen, occasionally touching the sexual organs of both inflorescences. Thus, they also contributed to pollination but are not as important as hoverflies (Table 4).

**Fig. 5:**
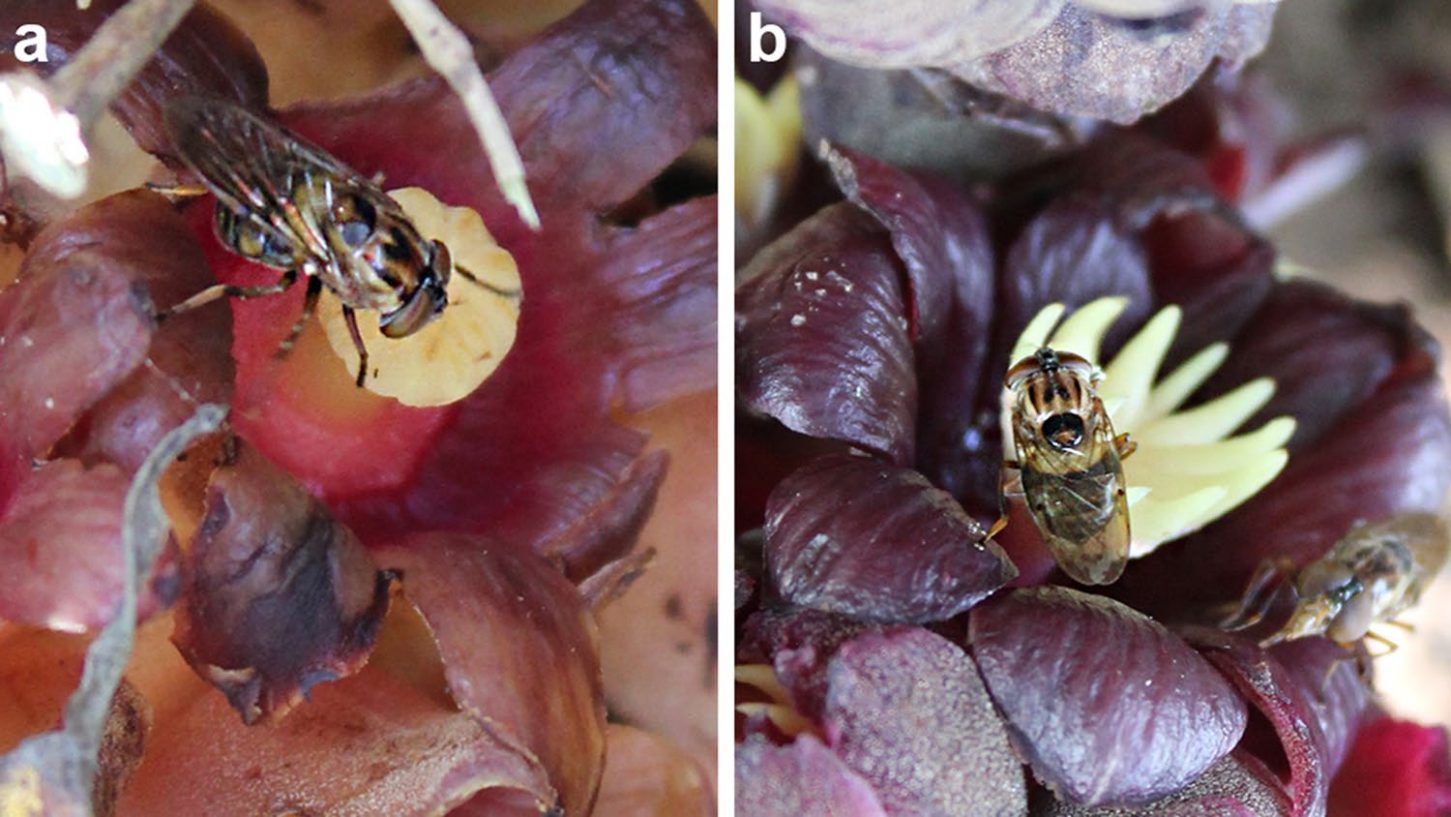
Pollinators of *B. americanum* in the studied population. **a** *Copestylum* sp. 1 landing on the stigma of female flowers. **b** *Copestylum* sp. 1 landing on connective appendages of male flowers, using them as perch to move around the flower

Finally, *Copestylum* sp. 1 hoverflies were the most frequent visitors in both male and female inflorescences (Fig. 4) during both years of study. They carried large amounts of pollen, exclusive to *B. americanum*, which was transported from male to female flowers. Also, *Copestylum* sp. 1 were the insects with the most records and visited the most flowers and inflorescences per cluster (Tables 3 and, 4). These floral visitors consistently touched the sexual organs. In male flowers, hoverflies landed on staminal connective appendages and used them as perches to move underneath the anthers and the rest of the flower (Fig. 5a). When they reached the female flowers, they landed on the stigma and began to move along the perigone of the flower (Fig. 5b). Once the hoverflies landed on stigmas or connective appendages according to sex, they moved to visit different flowers along the same inflorescence and spent less time on female flowers than on male ones. During most of their visits, we noticed that hoverflies could remain for more than 30 min resting on the perigone of the male flowers after foraging on the anthers. Therefore, given the amount of pollen on the body, the visitation frequency on both types of flowers, the constant contact with sexual organs, and the higher PII, hoverflies were the most important pollinators of *B. americanum* in the study area.

### Role of staminal appendages in pollination

**Fig. 6:**
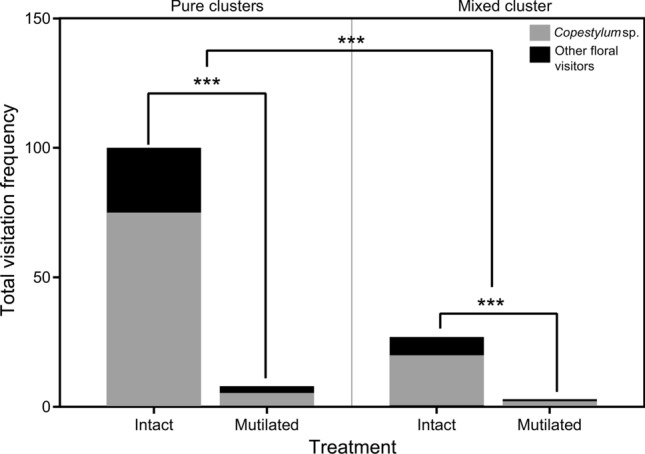
Visits to intact (control) and mutilated male flowers from mixed and pure clusters resulted in the connective appendages experiments. Pure clusters: patch with all inflorescences with the same condition (either intact or mutilated). Mixed cluster: patch with both mutilated and intact inflorescences randomly distributed. Asterisks show differences in the visitation frequency between treatments and clusters at *p* < 0.001

The frequency of visits differed between mutilated and intact (control) male flowers (χ^2^ = 114.56, df = 1, *p* < 0.0001). In the absence of connective appendages, pollinators approach clusters but do not land on flowers. The visitation frequency was lower in mutilated flowers, which was observed in both clusters (pure and mixed). However, the mixed cluster was less visited than the pure intact cluster (χ^2^ = 46.79, df = 1, *p* < 0.0001) indicating that mutilated flowers affected the visits to the rest of the inflorescences within the cluster (Fig. 6). Also, visitors seem to discern between mutilated and intact flowers because the visits were more frequent in the control than in the mutilated inflorescences within the same cluster. Visiting hours were mainly daytime, and the visits were consistent with the pollination observations where hoverflies were noticeable as the most frequent visitors in both treatments and clusters (Fig. 6).

## Discussion

### *Bdallophytum americanum* population

*Bdallophytum americanum* growth showed re-emergence of same-sex inflorescences over the years. The inflorescence emergence occurs gradually during blooming, so the variation between the number of exophytes between years and between clusters depends on the moment they are observed. The same pattern of re-emergence according to sex was reported for other species of Cytinaceae, such as *Cytinus hypocistis* (De Vega et al. 2007) and *C. sanguineus* (Hobbhahn and Johnson 2015). This feature has also been observed in *Pilostyles thurberi,* another endoparasite of the family Apodanthaceae (Ortega-González et al. 2023). In *C. hypocistis,* the re-emergence of exophytes has been followed over 5 years, indicating that this species has perennial plants (De Vega et al. 2007).

*Bdallophytum americanum* is a dioecious species whose male and female plants differ during emergence. The male inflorescences emerge before the female ones. This asynchronous emergence could affect reproduction. Female inflorescences take more time to appear as they need more resources to reproduce (Conn and Blum 1981). While male inflorescences can be advantageous since they can compete for pollinators with other species growing on the same site and keep floral visitors close to the area, taking advantage of their learning skills and awareness of the resource offered by *B. americanum* flowers (Purrington and Schmitt 1998; Weiss 2001).

### Floral movements and their implications in pollination

Here, we described for the first time the floral movements in a species of Cytinaceae. In *B. americanum*, the perigone movements occur during the morning and stop at noon, when the flowers are fully open, exposing the sexual organs. Stigmas from female flowers of *B. americanum* remain receptive for three days, while pollen grains from male flowers are viable for up to five days. Thus, all-day exposure to sexual organs allows a wide range of pollinators to visit flowers at different schedules favouring the reception and collection of pollen (Ganie et al. 2021; van Doorn and van Meeteren 2003). On the other hand, the lack of movements that cause the flowers to close can be disadvantageous since sexual organs are exposed to florivores, robbers, or damage (van Doorn and van Meeteren 2003). In this sense, floral movements are essential to flower and pollination ecology (Henning et al. 2018; Sibaoka 1969).

Additionally, a novel floral movement is described here for the family. The apical staminal connective appendages move synchronously in all stamens and parallel with the direction of the perigone when it opens. This parallel movement of stamens and perianth is described for other angiosperms where anthers and filaments are the moving structures (Henning et al. 2018; Zhang et al. 2019). In *B. americanum*, the staminal structures that move are the connective appendages that have an important role during pollen collection. In the first two days of anthesis, when the staminal appendages are completely extended or “open”, the pollen is at its maximum viability, favouring viable pollen presentation. When the connective appendages begin to “close” or return to their initial position on the third day of anthesis, the pollen viability decreases abruptly. This match between changes in pollen viability, the extended staminal appendages, and pollination is discussed in depth in the following sections.

### Floral visitors and pollination

The pollination patterns can vary depending on the habitat composition, availability of resources, and surrounding communities of organisms (Evans et al. 2017). A previous study on the pollination biology of *B. americanum* (formerly *B. bambusarum*) described how both male and female flowers produce nectar; however, it is more concentrated in female flowers (García-Franco and Rico-Gray 1997). The presence of floral rewards in both morphs explains the floral visits to male and female flowers, but in the studied population of *B. americanum*, all flowers lacked nectar. Still, hoverflies carried pollen exclusively from *B. americanum* and consistently touched the stigma. Thus, pollination can occur despite the lack of nectar. Despite the differences in the floral rewards offered by *B. americanum* to pollinators in different populations, dipterans with carrion behaviour are maintained as pollinators (García-Franco and Rico-Gray 1997). This pattern is also observed in other endoparasites such as *Sapria ram* (Rafflesiaceae), where the pollinators in different populations are consistently carrion flies, indicating a specialized interaction (Bänziger and Pape 2004; Pape and Bänziger 2000).

Studies of the floral ecology of other sapromyophilous endoparasites, such as the Rafflesiaceae species, have described the floral scent as one of the main attractants to pollinators (Zain et al. 2020). The foetid smell of the sapromyophilous species is strongly related to attracting carrion flies (Chakraborty and Adhikary 2018). Although syrphids are known to feed on pollen and nectar, members of the subfamily Eristalinae (where *Copestylum* belongs) have been reported as helpful in forensic entomology due to their consumption of carrion material (Martins et al. 2010). Specifically, hoverflies of the genus *Copestylum* were found in rodent carcasses, indicating that these organisms exhibit carrion behaviour (Moretti et al. 2008). Thus, although the potential pollinators are hoverflies of the genus *Copestylum* rather than the expected carrion flies, the carrion pollination is maintained given the carrion foraging behaviour of *Copestylum* sp. 1 in *B. americanum* flowers. Visits of *Copestylum* to female flowers are shorter and less frequent than to male ones. This might be because the female flowers do not produce rewards such as pollen or nectar, nor do they display large appendages like the male ones. Other attractants, such as scents may be conspicuous enough to attract pollinators, mostly in cases of sapromyophily, in which foetid smells are crucial for pollination to occur (Jürgens and Shuttleworth 2015). As hoverflies of the genus *Copestylum* can be attracted to carrion, pollination in this population appears to occur by deception. The female flowers do not offer a reward but expel a strong unpleasant smell. To confirm deceit pollination in this nectarless population it is necessary to study the emission of volatile organic compounds (VOCs) and the morphoanatomy of flowers to assess the functionality of nectaries.

Sapromyophilous traits are exclusive of the genus *Bdallophytum* within the Cytinaceae (Rios-Carrasco et al. 2022a, 2022b). Despite those floral traits such as dark floral colour and foetid smell resembling decaying material, pollination by carrion flies has only been reported in one population of *B. americanum* (García-Franco and Rico-Gray 1997). The other *Bdallophytum* species *B. andrieuxii* and *B. oxylepis* are pollinated by butterflies, and stingless bees, respectively (Rios-Carrasco et al. 2022a, 2022b), in addition to the present study in which *B. americanum* flowers are pollinated by hoverflies. Although butterflies, stingless bees, and hoverflies are not typically classified as carrion pollinators like certain groups of flies or beetles (Jürgens and Shuttleworth 2015), their carrion behaviour includes them as carrion pollinators. Thus, it is necessary to discuss based on the background information on visitor behaviour to better understand their role as pollinators.

### Staminal connective appendages as landing platforms

Flowers of *B. americanum* display a set of signals to attract pollinators, including the unpleasant smell, the dark colour of the flowers, and the long and apical connective appendages. Previous studies on *B. americanum* pollination did not address the role of apical connective appendages (García-Franco and Rico-Gray 1997). Thus, here, we describe for the first time their role in pollination. Our results indicated that the visitation frequency decreases sharply without the connective appendages in all flowers within a cluster. It is necessary to consider that the mutilation of staminal connective appendages could cause the releasing of VOCs associated with plant defence against herbivory affecting the visitation frequency (Suetsugu et al. 2022). To confirm this, studies are needed to assess the VOCs emission under herbivory conditions.

In a second scenario where both treatments were on the same patch, the visitation frequency was less in mutilated than in the intact flowers. Moreover, almost all visits were performed by hoverflies. The preference for intact flowers within the same cluster indicates a pollinator’s decision-making. Thus, there is a possibility that staminal appendages function as a visual attractant. According to the behaviour recorded for hoverflies (the most important pollinators in this population), they landed and perched on the staminal appendages. In *Mitella pauciflora* (Saxifragaceae), a fly-pollinated species, the petals have long filiform structures serving as landing platforms. In their absence, the visitation rate decreases (Katsuhara et al. 2017) as in *B. americanum*. As we mentioned previously, the “opening” of connective appendages matches the maximum pollen viability and is the structure where the hoverflies land. Studies on stamen movements mentioned that motion is an adaptation that facilitates pollen removal (Abdusalam et al. 2021). In *B. americanum*, apical connective appendages enhance the removal of viable pollen, given the match between the highest pollen viability and the fully extended connective appendages (that form the landing platform). Thus, the connective appendages in *B. americanum* can serve as a landing platform that improves pollen presentation, collection, and transfer favouring reproduction (Henning et al. 2018; Lawson and Rands 2018; Tan and Tan 2018).

Additional floral appendages have been related to fly pollination (Faegri and van der Pijl 1979). Some fly-pollinated species have flowers with filiform appendages with different roles in pollination. For instance, in Rafflesiaceae, filiform structures resembling a filter to stamens allow the flies to traverse to the anthers but avoid the entry of other non-pollinators (Bänziger 1995). In a recent study on *Arisaema urashima* (Araceae), authors demonstrate that the long filiform appendage on inflorescences plays an important role in pollination carried out mainly by fungus gnats; in the absence of the appendage, visitation frequency decreases affecting the fruit and seed set (Suetsugu et al. 2022). In this study, we explore the role of staminal connective appendages during pollination, but their importance related to fruit and seed formation remains to be explored. Suetsugu et al. (2021) hypothesize that the long appendage plays a role in the emission of VOCs as pollinator attractant thus, in their absence, the pollinators did not approach inflorescences (Suetsugu et al. 2022). We cannot discard that staminal appendages of *B. americanum* male flowers could participate in the release of volatile attractants. It is necessary to confirm if staminal appendages emit attractive VOCs.

The pollination of a nectarless population of *Bdallophytum americanum* was studied, emphasizing the functional role of connective appendages. Our results show that pollination by dipterans is maintained in *B. americanum* despite lacking nectar. We described a novel type of movement in the staminal connective appendages of the androecium that coincides with the changes in pollen viability. Potential pollinators were bees, beetles, flies, and hoverflies as they visited male and female flowers carrying pollen grains on their bodies. Hoverflies of the genus *Copestylum* stood out as the most important pollinators in the studied population of *B. americanum*. The connective staminal appendages served as a landing platform for hoverflies. This landing platform appears crucial for pollinator positioning and the collection of viable pollen, but it cannot be discarded as a visual attractant. The form-function relationship of specialized structures must be studied in detail to better understand the animal-plant interactions, particularly in the misunderstood endoparasites.
