# Correction to: The floral biology and the role of staminal connective appendages during pollination of the endoparasite *Bdallophytum americanum* (Cytinaceae)

**DOI:** 10.1007/s10265-023-01481-5

**Published:** 2023-07-13

**Authors:** Sandra Rios-Carrasco, Daniel Sánchez, Pactli F. Ortega-González, Morayna F. Gutiérrez-Luna, Manuel Edday Farfán-Beltrán, María C. Mandujano, Sonia Vázquez-Santana

**Affiliations:** 1 Laboratorio de Desarrollo en Plantas, Departamento de Biología Comparada, Facultad de Ciencias, Universidad Nacional Autónoma de México, Ciudad de Mexico, 04510 México; 2 Posgrado en Ciencias Biológicas, Universidad Nacional Autónoma de México, Ciudad de Mexico, 04510 México; 3 Departamento de Botánica y Zoología, Centro Universitario de Ciencias Biológicas y Agropecuarias, CONACYT–Laboratorio Nacional de Identificación y Caracterización Vegetal, Universidad de Guadalajara, Zapopan, 44171 Jalisco México; 4 Posgrado en Ciencias Biológicas, Instituto de Ecología, Universidad Nacional Autónoma de México, Coyoacán, Ciudad de Mexico, 04510 México; 5 Laboratorio de Genética y Ecología, Departamento de Ecología de la Biodiversidad, Instituto de Ecología, Universidad Nacional Autónoma de México, UNAM, Ciudad de Mexico, 04510 México

**Correction to: Journal of Plant Research**

10.1007/s10265-023-01466-4

In the original publication of the article, figure 4 was published incorrectly. The correct figure is provided in this article.

**Figure:**
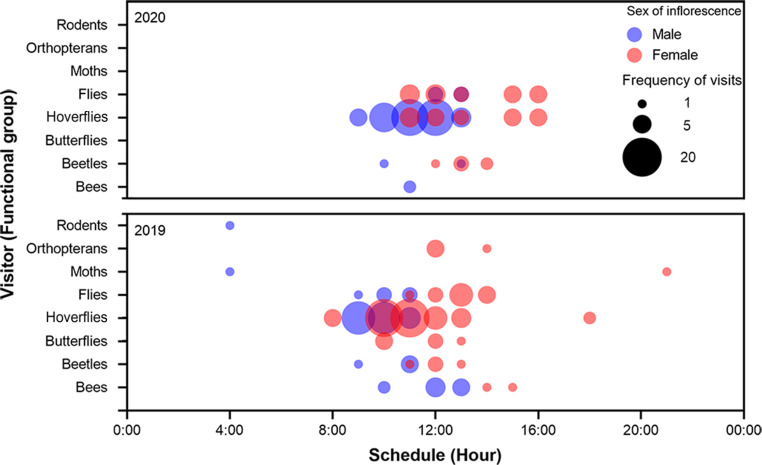
Fig. 4

